# Normativity of Predictions: A New Research Perspective

**DOI:** 10.3389/fpsyg.2019.01710

**Published:** 2019-07-23

**Authors:** Michał Piekarski

**Affiliations:** Institute of Philosophy, Cardinal Stefan Wyszyński University in Warsaw, Warsaw, Poland

**Keywords:** predictive processing, normativity, active inference, uncertainty, mechanism, environment, content, casuality

## Introduction

One of the most interesting philosophical aspects of predictive processing (PP) is the normativity of predictive mechanisms and its function as a guide of action. In my opinion this framework provides us with good tools to describe and explain the phenomenon of normativity. It is possible to justify the thesis that explanations in the PP approach are normative in nature. They are like that because predictive mechanisms themselves are normative. By normative function of prediction I understand a feature of prediction which is constitutive (Bickhard, 2003) for action control as well as for the structure and content of the world model that is internal to a given cognitive system. They are normative in the sense of possibly being wrong (Bickhard, 2015a,b, 2016). Normative are also some properties of the environment. Both those factors are crucial for content and truth-value of representations. With no normativity, there is no error and it is hard to explain the possibility of misrepresentations. It means that predictions are also normative for action because they can be true (more probably in the Bayesian manner) or false (less probably in the Bayesian manner).

## Predictive Processing Framework

In the PP framework (Friston, 2010; Clark, 2013, 2016; Hohwy, 2013), the main function of the brain (understood as a multilevel, hierarchical, and generative model) is to minimize prediction errors, i.e., any potential discrepancies between information from sensory input and expectations related to the source and nature of such information. This function is of key importance for the organism because, according to the PP framework, all perception serves the aim of ensuring that the organism operates efficiently in its environment: the brain keeps creating statistical predictions of what happens in the world. It predicts the current and future forms of information reaching the brain through sensory modalities. The predictions are hierarchically arranged and created at individual levels of the model. Thus, estimates made at different hierarchical levels relate to predictions present at other levels. More precisely, predictions impose a top-down structure on the bottom-up flow of information coming from the senses. Prediction errors are used by the model (at each level) to correct its current estimate of the input signal and generate the next prediction. The aim of low-level predictions is to clarify the spatial and temporal dimensions of incoming information. Predictions at higher levels of the model are more abstract. This framework suggests that the brain copes with making predictions by continuously estimating and re-estimating its own uncertainty (Clark, 2016, p. 57). What does it mean?

Estimations of uncertainty alter the impact of prediction errors. This function is directly related to so-called attention, i.e., a means of balancing the relations between top-down and bottom-up influences via precision, which is a measure of their estimated certainty. The greater the precision, the lesser the uncertainty (Friston, 2010). In this approach, “uncertainty” means that a given piece of information may be described through probability distribution. Here, the best possible prediction is made by applying Bayes' Rule which describes the probability of a given hypothesis (prediction) based on the brain's prior knowledge of conditions that might be related to the incoming sensory signal (e.g., Hohwy, 2013; Harkness and Keshava, 2017). The Bayesian approach offers a rational solution to the problem of how the brain updates the generative model (together with the internal model of the world) on incoming sensory signals and the question of the hidden causes behind these signals.

## Minimization of Free Energy

Minimization of free energy (in the informational-theoretical sense) consists in changing internal representations of the model in such a way as to approximate the posterior density of the causes of sensations (Friston et al., 2010). This means that free energy is minimized when there is a change in predictions about the sources of statistical information obtained from sensory input. The change can be achieved by either (1) altering the properties of the model (changing adopted predictions)—perceptual inference; or (2) changing the environment through active inference, i.e., an action that modifies the state or causal structure of the world; thereby generating new sensory information. Perception reduces free energy by changing predictions, whereas action achieves the same by changing the information reaching the model. Therefore, the biological systems described by Friston should be interpreted in terms of active agents who minimize prediction errors with the use of a probabilistic generative model. It follows that the minimization of prediction errors serves a normative function in relation to the agent. First, it maintains a homeostatic balance; second, it obliges the agent to make predictions about the state of the world in order to learn the unknown parameters responsible for its motion by optimizing the statistical information coming from sensory input (Friston et al., 2010, p. 233).

This normative and abductive aspect of policy selection plays a key role for the interpretation of free energy minimization as approximate Bayesian inference or self-evidencing (Hohwy, 2016). In other words, it speaks to the fact that uncertainty-reducing policies have to be selected via a process of Bayesian model selection. This in turn rests upon the capacity to entertain counterfactual hypotheses like “what would happen if I did that.” Hence, active inference and PP goes beyond homoeostasis and, possibly, becomes a purely personal inference (Seth, 2015)^1^.

## Normative Predictions

The issue of normativity is crucial for PP framework. Minimization of prediction errors directly implies “low-level” biological normativity. Friston connects it with the free energy principle (FEP) which suggests that all biological systems are driven to minimize information-theoretic “free energy,” which he understands as the difference between an organism's predictions about its sensory inputs and the sensations it actually encounters (Friston, 2010; Friston et al., 2012a). In this sense, FEP is a normative theory of action and perception, because it provides a well-defined objective function (variational free energy) that is optimized both by action and perception. The normative aspect of FEP is complemented with PP approach as a neuronally plausible implementation of this function (Schwartenbeck et al., 2013, p. 1). At higher levels of the model, normativity may be linked with (1) patterns of neural excitations based on predictions and (2) the role played by predictions in decision-making and action-control processes, among others, to minimize uncertainty in the environment^2^. The latter functionality is specifically important for our reflections and should be primarily related to active inference.

In Bickhard's opinion minimization of uncertainty alone is not enough to talk about normativity, because it is rather a “supposed consequence of the effect of prior evolutionary selection.” This means that the functioning of an organism is explained based on the existence of factual and casual conditions which minimize discrepancies between internally generated predictions and signals from sensory inputs (Bickhard, 2016, p. 264). In this sense, it is difficult to explain how and why an organism seeks value or avoids harm. It can be said that it does so for reasons of evolution or training, but this can easily be countered with the objection of the Dark Room Problem (Friston et al., 2012b; Sims, 2017; Klein, 2018). Friston et al. (2009) claims that highest level expectations are “built-in” to the organization of the whole organism. However, the adoption of such a hypothesis does not make it possible to finally explain the “normative” difference between successful action and some kind of error or “mistake,” because, from the point of view of the organization of the system, all such processes are just casual and factual.

In PP approach predictions and expectations are in some sense normative because of their key role in minimizing prediction errors. However, as Bickhard emphasizes, they only involve actual and causal processes. The key issue, therefore, is to differentiate relations between predictions and actions that are not only causal but also normative. Following Bickhard, it must be stated that to explain the normativity of functions we must demonstrate how this normativity emerges from the natural organization of the organism. By this I mean that it is necessary to refer not so much to the structure of a given system as to its actual interactions with the environment. Therefore, the normativity of prediction is less determined by its functional role in the generative model and the selection and management of actions than by its reference to relevant properties of the environment which, according to some researchers (e.g., Bruineberg and Rietveld, 2014; Bruineberg, 2017; Piekarski and Wachowski, 2018), is already structured. Due to the fact that the world is already “pre-structured,” it may present to the organism certain values of reward or punishment which cannot be reduced to log-evidence or negative surprise (Friston et al., 2012a).

Based on the hypothesis it has formulated, the cognitive system takes relevant action which is supposed to interfere with the causal structure of the world in a way that will make the hypothesis or prediction probable or true (Clark, 2016, p. 116). In this sense, a relevant prediction serves a specific normative function which should be understood in two ways: as primary normativity; and as normativity of mechanism.

The research conducted here hinges upon primary normativity. A pair of “prediction—active inference (action)” can be treated as a kind of conditional of the form “z*If* ‘prediction' (condition) *then* ‘action' (result).” The relationship between prediction and action, however, is not a typical causal relationship that we can write symbolically as “*If* A, *then* B,” but the motivational relation “*If* A, *then* B, C *or* D, *but not* E, F *or* G.” This still raises the following question: “why is dependence on normative predictions normative (functional) but not causal?” The full answer to this question will only be possible if we bear in mind that this dependence is constitutive for the interaction between an organism and its environment, which means that, on the one hand, it cannot be reduced to the structure of the organism or a cognitive system “armed” with a generative model and simple mechanisms of reinforcement or unsupervised learning (Friston et al., 2009; Korbak, in preparation), and on the other hand, more importantly, it allows to make an error that will be significant from the point of view of the organism and not only the external observer assessing it (Bickhard, 2016, p. 263). In other words, predictions are normative because they refer not only to the need to minimize prediction errors or uncertainty, but also to the individual beliefs^3^ or motivations that arise in the face of specific possibilities for action (affordances) that the environment offers to an individual organism. From this perspective, a possible error or wrong representation is of normative importance to the organism and not merely a potential result of specific causal processes. Normative involvement of predictions is also constituted by how they shape the causal transitions between contentful states and structured environment [in such a way that they accord to a normative Bayesian rule (Shams et al., 2005;Kiefer, 2017)].

For example: if I predict it will rain, then this prediction obliges me (Friston, 2010, p. 233) to take some action (which is not entirely arbitrary but determined by the nature of the prediction): I can stay at home, order a taxi or take an umbrella, but the prediction does not necessarily determine activities such as going to bed or watching TV (i.e., it might be difficult to justify these actions by referring to the prediction that it will rain as their reason What I do depends also on my beliefs, desires, or goals, which are relativized and conditioned by the specific properties of the world. In this sense, predictions should be considered as normative. It means that actions are selected based on some conditional potentiality and relations. “Such conditional relationships can branch—a single interaction outcome can function to indicate multiple further interactive potentialities—and they can iterate—completion of interaction *A* may indicate the potentiality of *B*, which, if completed, would indicate the potentiality of *C*, and so on” (Bickhard, 2009, p. 78)^4^.

It is important to add that the cognitive system still predicts the form of sensory signals via active inferences (actions). Those depend on normative predictions which are at the same time verified by active inference. The dependence is not causal but functional (or normative, in my terms). How effective active inference is in minimizing prediction errors hangs on the selection of predictions and internal parameters of the model. The adaptive and cognitive success of an organism is the product of normative predictive mechanisms related to some aspects and features of the environment.

The normativity of prediction can be additionally justified as the normativity of a mechanism: a given mechanism is normative because it fulfills the conditions that must be met in order for a given action (cognitive or non-cognitive) to be affected. In other words, the statement that a given mechanism is normative simply means that it is possible for a given system *X* to be a mechanism for activity *Y* even though (e.g., at a given moment) *X* cannot perform *Y* (Garson, 2013). This means that predictions are primarily normative as well as embodied in a mechanism that is itself normative.

## Conclusion

In my opinion, the PP approach offers a brand-new framework for investigations into the problem of normativity. This possibility has been ignored in the current literature, but it is a legitimate object of further investigation. Further theoretical research is, therefore, warranted to determine the extent and nature of the interaction between predictive mechanisms and their normative functions.

## Author Contributions

MP reviewed the literature, developed the theoretical stance, wrote the manuscript and prepared to publication.

### Conflict of Interest Statement

The author declares that the research was conducted in the absence of any commercial or financial relationships that could be construed as a potential conflict of interest.

## Footnotes

^1^In these considerations I take the perspective of those researchers who claim that Friston's analyses are to some extent complementary with the PP approach (Bruineberg and Rietveld, 2014; Hohwy, 2015; Seth, 2015; Bickhard, 2016; Kirchhoff et al., 2018).

^2^Technically, the minimization of uncertainty corresponds to minimizing expected free energy through action or policy selection. In terms of information theory, uncertainty is the divergence between the predicted and preferred (sensory) outcomes (Friston et al., 2015).

^3^I use the notions of belief in the Bayesian manner as a probability distribution over some unknown state or attribute of the world. In this sense belief is a systemic prior with a high degree of abstraction, i.e. a high-level prediction concerning general knowledge about the world.

^4^It is important to refer here to the already mentioned concept of counterfactual hypotheses. The choice between these hypotheses is directly related to Bayesian inference to the best explanation and is crucially important for social cognition (Palmer et al., 2015).
